# Has the Child Dental Benefits Schedule improved access to dental care for Australian children?

**DOI:** 10.1111/hsc.13803

**Published:** 2022-03-25

**Authors:** Nicole Stormon, Loc Do, Christopher Sexton

**Affiliations:** ^1^ School of Dentistry UQ Oral Health Centre The University of Queensland Brisbane Queensland Australia

**Keywords:** access to health care, child health, health and social service policy analysis, policy research, primary dental care, public health policy

## Abstract

The Child Dental Benefits Schedule (CDBS) is an ongoing scheme administered through the Australian Government providing eligible children funding for clinical dental treatment. This study aimed to investigate the access of dental services across children's early childhood and examine whether the CDBS has improved access to dental care. The longitudinal study of Australian children is an ongoing cross‐sequential cohort study with a representative sample of Australian children recruited in 2004. Birth (0–1 year) and kindergarten (4–5 years) cohorts were recruited through Medicare enrolment information at baseline and were representative of the Australian child population. Population‐weighted longitudinal mixed effects Poisson models with individual identifiers as a random effect were used to assess the effect of Medicare dental schedules on reported dental attendance. Prior to the implementation of the CDBS for both cohorts, the birth cohort reported the lowest attendance rate at age 4–5. The introduction of the CDBS increased the rate of dental attendance for the low household income group by 8% (95% CI: 1%, 15%) after adjusting for confounders. The model provides evidence that dental attendance increased with age and the Indigenous population have 31% (95% CI: 4%, 55%) lower attendance rate after adjustment. The increase in reported access to dental services and favourable visiting patterns in low‐income households during the operation of the CDBS provides some evidence that the schedule's primary aims to improve access to care in the child population are being met. Access to healthcare is multifaceted and the underutilisation of the schedule in the population warrants review of the schedule performance using other patient‐centred indicators.

**What is known about this topic?**

- The Child Dental Benefits Schedule (CDBS) is a government‐funded schedule aimed at improving access to dental care for the Australian population.
- It has been reported to be underutilised and no studies have investigated if the schedule is meeting the policy goals.

**What this paper adds**

- The CDBS is improving reported access to dental care for children in low‐income households.
- Innovation and policy reform must be explored to improve access for Aboriginal and Torres Strait Islander children and younger access for all income levels.
- Whilst the structure and administration of the CDBS has improved access for sections of the community, there are underserved populations that require urgent additional support to access dental services.

## 1 INTRODUCTION

Dental decay (caries) is the most common chronic disease and there have been increasing calls for action (Peres et al., 2019). Dental caries is not simply caused by poor tooth brushing behaviours and sugary diets, but complex interlinked social and system‐level factors (Watt, 2002). By early adolescence, more than half of Australian children experienced dental caries, and the burden of disease is inequitably distributed for disadvantaged and marginalised children (Stormon et al., 2019). Untreated dental caries results in pain, school absenteeism and lower quality of life for children (Ghorbani et al., 2017). Untreated caries also results in complex and costly treatment needs as well as preventable hospital admissions (Alsharif et al., 2016).

Australia's dental care system is a mixed healthcare model, where individuals can access care through either public or privately operated services. In the private model, individuals pay on a fee‐for‐service basis and can purchase private health insurance to cover part of the expenses (Lam et al., 2012). The public sector is funded by the Commonwealth and State and Territory Governments and generally low socioeconomic groups and children are eligible for subsidised care (Queensland Government, 2019; Victoria State Government, 2020). Over half (56%) of Australian children reported accessing dental services in the private sector (Do & Spencer, 2016). Approximately 40% of the population hold private insurance for dental treatment, yet even these individuals often pay substantial gap fees (Lam et al., 2012). This unavoidable financial burden explains why over a third of Australians either postpone or evade dental treatment due to cost (Ellershaw & Spencer, 2011).

In 2008, the Australian Commonwealth Government introduced the Medicare Teen Dental Plan (TDP) for 12‐ to 17‐year olds from low‐income families. Eligible teenagers received an annual voucher of around $150 (AUD) towards the cost of an annual preventative dental check‐up (Australian Research Centre for Population Oral Health, 2011). Underutilisation of the TDP in vulnerable populations contributed to a review of the schedule and its subsequent cessation on 31 December 2013. In 2014, the TDP was superseded by a new dental health reform package; the Child Dental Benefits Schedule (CDBS) aimed at improving access to a wider range of dental services and treatment (Australian National Audit Office, 2015). CDBS is an ongoing schedule administered through the Australian Government medical insurance schedule Medicare. Eligible 2‐ to 17‐year‐old children can access $1000(AUD) of dental treatment over 2 calendar years in the public or private sectors (Australian National Audit Office, 2015). The schedule objective was to ‘*Improve access to dental services for children*’ and ‘*help children develop good oral health habits early in life and help to arrest the increase in child dental decay*’. (Australian National Audit Office, 2015) In the most recent statutory review of the schedule, $1.4 billion worth of benefits was paid from implementation to 2018 in the schedule (Commonwealth of Australia, 2019). However, this was reported to be 41% lower than the projected expenditure on the schedule (Australian National Audit Office, 2015). The Australian Federal Budget 2021–2022 increased the schedule budget by $7.3 million over 4 years to include children less than 2 years of age.

The Department of Human Services notified approximately 3.1 million children in 2014 and 2.9 million children between January and June 2015 of their eligibility under the schedule (Australian National Audit Office, 2015). An audit of the schedule in 2015 found that less than 30% of the eligible child population were utilising the program (Australian National Audit Office, 2015). The schedule audit recommended the administration of the schedule be reviewed and barriers to utilisation investigated (Australian National Audit Office, 2015).

Since the publication of the audit of the CDBS, five studies have been published investigating CDBS utilisation. A study by Putri et al. (2019) reported a decline in service utilisation by 16.3% after the 1st year of the CDBS (Putri et al., 2019). Another study found that schedule utilisation rates were similar in Indigenous and non‐Indigenous Australians; however, less preventive services were claimed in Indigenous children (Orr et al., 2021). Mothers with mental health conditions and poor health behaviours (such as smoking) were found to be predictors of non‐utilisation of the schedule in the Longitudinal Study of Australian Children (Nguyen et al., 2021).

The multi‐billion‐dollar schedule continues to be supported in the Commonwealth budget each year; however, the question still remains, does the Child Dental Benefits Schedule increase access to dental care for Australian children? The primary performance indicator of the schedule was a goal of 2.4 million eligible children accessing the schedule in the first 2 years since implementation and evidence suggests that this is not being met (Australian National Audit Office, 2015; Putri et al., 2019). Additionally, an audit of the schedule stated that these performance indicators do not *provide a complete picture of the performance of the CDBS in meeting program objectives—improving access to dental services for children and improving population‐wide oral health* (Australian National Audit Office, 2015). There is a lack of evidence to demonstrate that the schedule has resulted in an increased access to dental services for the Australian child population. With evidence of underutilisation of the schedule, further research is warranted to explore if the primary aim of the schedule is being met. This study, therefore, aims to assess the impact of the implementation of CDBS on access to dental care in the Australian child population, particularly children of low socioeconomic backgrounds who are the target of the schedule.

## 2 METHODS

### 2.1 The longitudinal study of Australian children

The LSAC is a cross‐sequential dual cohort study run biennially since baseline data collection in 2004 (referred to as wave one). At wave one, 5,107 children participated from the birth (B) cohort (aged 0–1 year) and 4,983 from the kindergarten (K) cohort (aged 4–5 years). Nine waves were available for use and Table S1 reports the sample size and response rate across the study waves. The study child's primary caregiver completed either a telephone or computer‐assisted questionnaire. Ethical approval for the LSAC was granted by the Australian Institute of Family Studies Ethics Committee. Further information on the LSAC study design, ethics approval numbers and how to access the dataset can be found in data user guides and technical reports published online (Australian Institute of Family Studies, 2019).

The study child's carer reported if dental service was used in the previous year and was collected in waves two and three for the K (6–7 years of age) and B (4–5 years of age) cohorts, respectively. The TDP was implemented on 1 July 2008 and ceased on 31 December 2013. The CDBS was implemented on 1 January 2014 and is ongoing. The B and K cohorts were 10 and 14 years of age, respectively, when the CDBS was implemented (Table S1).

Sociodemographic and socioeconomic variables included sex, Australian state of residence, Aboriginal and or Torres Strait Islander (herein respectfully referred to as Indigenous) status, Australian statistical geography standard (ASGC) (major city, inner regional, outer regional and remote/very remote), household income (recoded into tertiles) and Socio‐Economic Indexes for Areas Advantage/ Disadvantage (SEIFA) (recoded into tertiles). Carer‐reported receipt of the Family Tax Benefit, Parenting Payment Partnered or Single was reported as a proxy measure for CDBS eligibility as actual eligibility was not linked to the LSAC data.

### 2.2 Statistical analysis

Stata 14.2 (College Station, TX) was used for data analysis and figures were created using the ggplot2 (v.3.3.3) and ggpubr (v.0.4.0) packages in RStudio (Boston, MA).

Survey commands (svyset) were used to account for stratification by areas within states, clustering by postcodes and weighting due to potential non‐response. Population weights were applied, so analysis is representative of the Australian Bureau of Statistics‐estimated resident population counts of children in March 2004 for children 0 and 4 years of age, respectively. Further information on the survey design and the calculation of population weights is available from the LSAC technical reports (Australian Institute of Family Studies, 2019).

Descriptive analysis of participant demographics and carer‐reported dental service use over time were reported by weighted population percentage and 95% confidence interval (CI). The percentage of carer‐reporting dental service use in the previous year was presented in a cohort table. Cohort effects were observed by examining intercohort changes (reading down the columns). Period effects were examined by comparing the same age group at one time point, with data at another time point (reading across the rows).

Responses to sequential pairs of surveys were used to categorise children's dental visiting patterns as two surveys reporting visits (adequate), only one survey reporting visits (fair) and no reported visits in sequential surveys (poor).

Unweighted and weighted longitudinal mixed effects Poisson models with individual identifiers as a random effect were used to assess the effect of government schedules on dental attendance and dental visiting patterns. An interaction term between schedule and household income and SEIFA groups was included in the models as the government schedules were income tested. Models were adjusted for cohort, age, sex, Indigenous status and ASGS and are reported as prevalence rate ratios (95% CI). Marginal analysis of the fixed effects of the dental attendance model was used to graph the adjusted dental attendance percentage across age groups and stratified by income.

## 3 RESULTS

Table 1 reports the population‐weighted characteristics of the B and K cohorts. There were minor differences between the two cohorts across the demographic variables.

**TABLE 1:** Population‐weighted demographics of study children at baseline^a^ for the birth and kindergarten cohorts

	Birth		Kindergarten	
	4–5 years		6–7 years	
	2008		2006	
	%	(95% CI)	%	(95% CI)
Sex				
Male	51.1	(49.5, 52.8)	51.3	(49.7, 52.8)
Female	48.9	(47.2, 50.6)	48.7	(47.2, 50.3)
State of residence				
NSW	32.7	(30.9, 34.4)	33.5	(31.7, 35.3)
VIC	25.4	(23.7, 27.2)	25.1	(23.3, 27.0)
QLD	20.2	(18.4, 22.1)	19.7	(18.1, 21.5)
SA	6.9	(6.0, 7.9)	6.7	(5.8, 7.7)
WA	9.7	(8.6, 10.9)	9.8	(8.7, 11.1)
TAS	2.4	(1.7, 3.3)	2.5	(1.9, 3.2)
NT	0.8	(0.5, 1.4)	1.0	(0.5, 2.0)
ACT	1.9	(1.6, 2.2)	1.7	(1.4, 2.0)
Indigenous status				
Yes	4.9	(4.0, 6.1)	3.7	(3.0, 4.7)
Australian statistical geography standard				
Major city	68.5	(65.2, 71.7)	69.0	(65.4, 72.4)
Inner regional	19.9	(16.9, 23.3)	18.4	(15.1, 22.3)
Outer regional	10.3	(8.0, 13.1)	10.9	(8.3, 14.2)
Remote and very remote	1.3	(0.7, 2.4)	1.7	(0.8, 3.6)
Household income				
Low	38.0	(35.6, 40.4)	37.0	(34.8, 39.1)
Middle	32.1	(30.3, 34.0)	32.6	(31.0, 34.5)
High	29.9	(27.5, 32.4)	30.5	(28.1, 32.9)
Socio‐economic indexes for areas advantage/disadvantage^b^				
Low	36.0	(31.9, 40.4)	35.1	(30.4, 40.0)
Middle	33.0	(29.1, 37.1)	33.1	(28.7, 37.8)
High	31.0	(27.0, 35.4)	31.9	(27.3, 36.8)
Receives eligible payment^c^				
Yes	62.3	(59.7, 64.8)	65.3	(61.6, 66.0)

Note

Abbreviations: ACT; Australian Capital Territory, NSW; New South Wales, NT; Northern Territory, QLD; Queensland, SA South Australia, TAS; Tasmania, WA; VictoriaWestern Australia, VIC.

^a^ Baseline represents the first available wave in the LSAC where parents reported access to dental services in the previous year.

^b^ Low scores indicating disadvantage, high scores indicating advantage.

^c^ Eligible payments for the CDBS included Parenting Payment and the Family Tax Benefit.

The rates of dental attendance for the two cohorts increased during childhood and then peaked at age 12–13 for the B cohort and age 14–15 for the K cohort (Table 2). A similar trend was observed between children who received and did not receive eligible payments for the dental schedules (Figure S1). Prior to the implementation of the CDBS for both cohorts, the B cohort reported the lowest attendance rate at age 4–5 but increased to comparable rates as the K cohort at ages 6–7 and 8–9 years. After the introduction of CDBS, population‐level reported dental attendance rates increased for both cohorts. The increases in the percentage of dental attendance were small as the K cohort increased by approximately 2.5% and the B cohort increased by 5.5%. However, the dental attendance rate decreased in the final survey of both cohorts, whilst CDBS was still operational.

**TABLE 2:** Cohort table: Population‐weighted estimates children reporting dental service use in the previous year in the birth and kindergarten cohorts

Age (years)	2006		2008		2010		2012		2014		2016		2018	
	%	(95% CI)	%	(95% CI)	%	(95% CI)	%	(95% CI)	%	(95% CI)	%	(95% CI)	%	(95% CI)
4–5			33.7	(32.0, 35.4)										
6–7	59.4	(57.2, 61.5)			55.9	(53.7, 58.1)								
8–9			60.0	(58.1– 62.0)			62.3	(60.5, 64.1)						
10–11					61.1	(58.8, 63.4)			^C^ 65.4	(63.3, 67.4)				
12–13							^T^ 61.6	(59.5, 63.6)			^C^ 67.8	(65.8, 69.8)		
14–15									^C^ 64.1	(61.9, 66.2)			^C^ 64.1	(61.9, 66.3)
16–17											^C^ 60.1	(57.7, 62.3)		

Note

Cohort table: Age effect; reading diagonally down to the right, Cohort effect; reading down the columns, Period effect; reading across the rows.

Shading: Dark grey; B cohort, Light grey; K cohort.

Superscript: T; Teen Dental Plan, C; Child Dental Benefits Scheme.

**FIGURE 1:**
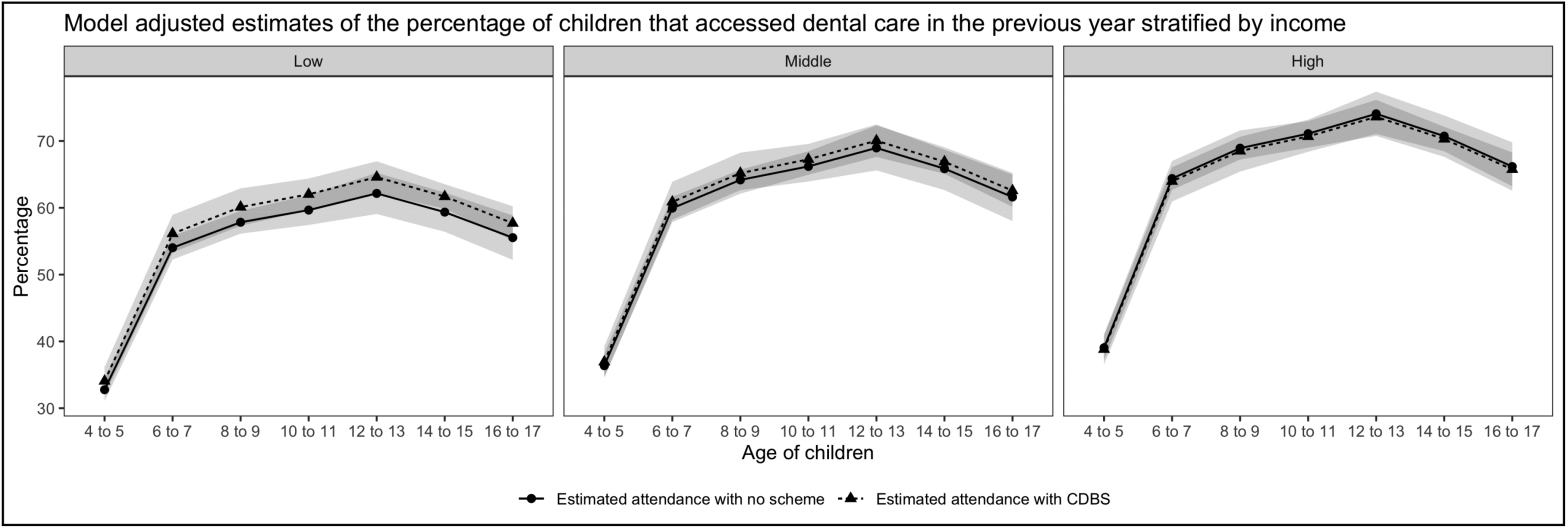
Model adjusted estimates of the percentage of children that accessed dental care in the previous tear stratified by income

The introduction of the CDBS increased the rate of dental attendance for the low household income group by 8% (95% CI: 1%, 15%) after adjusting for age, cohort, sex, SEIFA, Indigenous status and ASGS (Table 3). There was insufficient evidence that the dental attendance for the low‐ and middle‐income populations improved under the TDP schedule. The model provides strong evidence that dental attendance generally increased with age and the Indigenous population have 31% (95% CI: 4%, 55%) lower attendance rate after adjustment for other factors. The model‐adjusted estimates of dental attendance rates across ages for both cohorts were explored graphically by stratifying the populations into income categories (Figure 1).

**TABLE 3:** Adjusted unweighted and population‐weighted model of carer‐reported access to dental care

	Unweighted		Population weighted	
	PR	95% CI	PR	95% CI
Dental scheme				
*CDBS*	0.99	(0.94, 1.03)	0.96	(0.90, 1.03)
*TDP*	0.94	(0.88, 1.00)	0.93	(0.85, 1.01)
*No Scheme*	1	Ref.	1	Ref.
Income				
*Low*	0.84	(0.81, 0.87)	0.98	(0.92, 1.03)
*Middle*	0.93	(0.90, 0.96)	0.99	(0.94, 1.03)
*High*	1	Ref.	1	Ref.
Multiplicative effect of dental scheme on income categories				
*CDBS on Low Income*	1.05	(1.00, 1.09)	1.08	(1.01, 1.15)
*CDBS on Middle Income*	1.02	(0.98, 1.07)	1.04	(0.98, 1.10)
*CDBS on High Income*	1	Ref.	1	Ref.
*TDP on Low Income*	0.95	(0.88, 1.02)	0.96	(0.86, 1.08)
*TDP on Middle Income*	1.03	(0.96, 1.09)	0.98	(0.89, 1.06)
*TDP on High Income*	1	Ref.	1	Ref.
SEIFA╪				
*Low*	0.85	(0.81, 0.89)	0.91	(0.84, 0.98)
*Middle*	0.92	(0.88, 0.95)	0.96	(0.90, 1.01)
*High*	1	Ref.	1	Ref.
Multiplicative effect of dental scheme on SEIFA categories╪				
*CDBS on Low SEIFA*	1.03	(0.98, 1.08)	1.07	(1.00, 1.14)
*CDBS on Middle SEIFA*	1.00	(0.96, 1.04)	1.01	(0.95, 1.07)
*CDBS on High SEIFA*	1	Ref.	1	Ref.
*TDP on Low SEIFA*	1.01	(0.95, 1.08)	1.09	(0.98, 1.21)
*TDP on Middle SEIFA*	1.03	(0.96, 1.10)	1.05	(0.96, 1.15)
*TDP on High SEIFA*	1	Ref.	1	Ref.
Age (years)				
*4–5*	0.59	(0.55, 0.64)	0.60	(0.55, 0.66)
*6–7*	0.97	(0.91, 1.03)	1.01	(0.93, 1.10)
*8–9*	1.04	(0.98, 1.10)	1.12	(1.03, 1.21)
*10–11*	1.08	(1.03, 1.13)	1.12	(1.05, 1.19)
*12–13*	1.12	(1.07, 1.17)	1.15	(1.09, 1.22)
*14–15*	1.07	(1.03, 1.11)	1.10	(1.05, 1.15)
*16–17*	1	Ref.	1	Ref.
Cohort				
*Kindergarten*	0.99	(0.97, 1.02)	1.04	(0.99, 1.10)
*Birth*	1	Ref.	1	Ref.
Sex				
*Female*	1.06	(1.04, 1.08)	1.10	(1.04, 1.17)
*Male*	1	Ref.	1	Ref.
Aboriginal Torres Strait Islander status				
*Yes*	0.86	(0.79, 0.94)	0.69	(0.50, 0.96)
*No*	1	Ref.	1	Ref.
Australian Statistical Geography Standard				
*Major city*	1.06	(1.00, 1.13)	0.96	(0.81, 1.15)
*Inner regional*	1.05	(0.99, 1.13)	0.98	(0.80, 1.19)
*Outer regional*	1.07	(0.99, 1.16)	1.08	(0.90, 1.28)
*Remote/Very remote*	1	Ref.	1	Ref.

Note

Abbreviations: CDBS; Child Dental Benefits Scheme, TDP; Socio‐Economic Indexes for Areas Advantage/ Disadvantage; Teen Dental Plan, SEIFA.

There was evidence that the introduction of CDBS improved the favourable pattern of dental attendance for the population of children in the low‐income group after adjusting for other factors (Table 4). Indigenous children were 73% (95% CI: 49%, 86%) less likely to have adequate dental visiting habits than non‐Indigenous children after adjustment.

**TABLE 4:** Adjusted unweighted and population‐weighted model of carer‐reported favourable patterns^a^ of accessing dental care

	Unweighted		Population weighted	
	PR	95% CI	PR	95% CI
Dental scheme				
*CDBS*	1.00	(0.94, 1.06)	0.90	(0.82, 0.99)
*TDP*	0.98	(0.91, 1.05)	0.91	(0.83, 1.00)
*No Scheme*	1	Ref.	1	Ref.
Household income				
*Low*	0.74	(0.70, 0.79)	0.92	(0.84, 1.01)
*Middle*	0.92	(0.87, 0.97)	1.01	(0.94, 1.08)
*High*	1	Ref.	1	Ref.
Multiplicative effect of dental scheme on Income categories				
*CDBS on Low Income*	1.10	(1.02, 1.18)	1.18	(1.07, 1.30)
*CDBS on Middle Income*	1.00	(0.93, 1.06)	1.04	(0.96, 1.14)
*CDBS on High Income*	1	Ref.	1	Ref.
*TDP on Low Income*	0.99	(0.89, 1.09)	1.05	(0.93, 1.20)
*TDP on Middle Income*	1.03	(0.95, 1.12)	1.06	(0.96, 1.18)
*TDP on High Income*	1	Ref.	1	Ref.
SEIFA				
*Low*	0.80	(0.74, 0.86)	0.87	(0.76, 1.00)
*Middle*	0.88	(0.83, 0.94)	0.91	(0.83, 1.01)
*High*	1	Ref.	1	Ref.
Multiplicative effect of dental scheme on SEIFA categories				
*CDBS on Low SEIFA*	1.01	(0.93, 1.09)	1.08	(0.97, 1.21)
*CDBS on Middle SEIFA*	1.01	(0.94, 1.08)	1.03	(0.94, 1.13)
*CDBS on High SEIFA*	1	Ref.	1	Ref.
*TDP on Low SEIFA*	0.94	(0.85, 1.03)	1.00	(0.88, 1.14)
*TDP on Middle SEIFA*	1.01	(0.93, 1.11)	1.07	(0.97, 1.19)
*TDP on High SEIFA*	1	Ref.	1	Ref.
Age groups				
6–7	0.59	(0.54, 0.65)	0.54	(0.48, 0.61)
8–9	0.94	(0.87, 1.01)	0.93	(0.84, 1.03)
10–11	1.01	(0.95, 1.07)	1.02	(0.95, 1.09)
12–13	1.06	(1.00, 1.12)	1.05	(0.98, 1.13)
14–15	1.04	(0.99, 1.08)	1.05	(1.00, 1.11)
16–17	1	Ref.	1	Ref.
Cohort				
*Kindergarten*	0.98	(0.94, 1.03)	1.01	(0.88, 1.15)
*Birth*	1	Ref.	1	Ref.
Sex				
*Female*	1.09	(1.05, 1.13)	1.28	(1.13, 1.46)
*Male*	1	Ref.	1	Ref.
Indigenous status				
*Yes*	0.80	(0.69, 0.93)	0.27	(0.14, 0.51)
*No*	1	Ref.	1	Ref.
Australian Statistical Geography Standard				
*Major city*	1.14	(0.99, 1.32)	0.97	(0.74, 1.28)
*Inner regional*	1.11	(0.96, 1.28)	0.87	(0.65, 1.17)
*Outer regional*	1.15	(0.98, 1.34)	1.08	(0.86, 1.27)
*Remote/Very remote*	1	Ref.	1	Ref.

Note

Abbreviations: CDBS; Child Dental Benefits Scheme, TDP; Socio‐Economic Indexes for Areas Advantage/ Disadvantage; Teen Dental Plan, SEIFA.

^a^ Favourable pattern of accessing care was defined as reporting attendance for dental care over two sequential surveys. Both cohorts were restricted to their second survey and beyond.

## 4 DISCUSSION

This study explored the impact of two Medicare dental schedules by reported dental attendance in two cohorts of Australian children. The dual‐cohort design of the LSAC enabled the investigation of period and cohort effects on dental attendance and estimation of the effects of the schedules adjusted for confounders. Overall, this study found a marginal increase in reported dental attendance in low‐income groups in the CDBS schedule years.

The World Health Organization defines universal health coverage (UHC) as health services that can be accessed by the population when they are needed, without financial and physical access barriers (The World Health Organization, 2021). Robust financial supports are central in UHC and the CDBS is an ongoing Medicare schedule which targets this aspect of universal coverage for children. Access to healthcare, however, is multifaceted with the availability of services, approachability, appropriateness and acceptability other key domains in patient‐centred access to healthcare (Levesque et al., 2013). The increase in reported access to dental services and favourable visiting patterns in low‐income households during operation of the CDBS provides some evidence that the schedule's primary aim to improve access to care in the child population is being met (Australian National Audit Office, 2015). However, the middle‐income group in this study did not have evidence of increased reported access to dental care as a result of the CDBS despite a large proportion being eligible. Other performance indicators used to discuss UHC in dental care include access to clinically relevant care, access in early childhood and improved oral health outcomes in the population (Briggs, 2012; Reich et al., 2016). An audit of the CDBS after its 1st year of implementation recommended that performance indicators needed to be defined as simply monitoring the number of children accessing the schedule was not sufficient to demonstrate the performance of the schedule (Australian National Audit Office, 2015). The Department of Health agreed to this recommendation made in the audit; however, no action to this recommendation has been published since the audit of the schedule (Australian National Audit Office, 2015). Evidence‐based performance indicators should be investigated in future studies and the cost‐effectiveness of the schedule explored.

In successful UHC, it is essential to understand and address social inequities in access to health services (Reich et al., 2016). It is clear from this study that the Indigenous child population have substantially less‐reported utilisation of dental services. Even after adjustment for the CDBS, Indigenous children had 31% lower attendance rates than their non‐Indigenous counterparts. Another study investigating CDBS use in Indigenous children found overall use of this schedule similar to their non‐Indigenous counterparts (Orr et al., 2021). However, this result does not capture the difference in treatment modalities as Indigenous children had higher risk or requiring invasive dental treatment than non‐Indigenous children (Orr et al., 2021). This inequity has also been reflected in the distribution of dental disease with this population found to have an 18% higher rate of untreated caries than their non‐Indigenous counterparts (Do & Spencer, 2016). Access and utilisation of healthcare is multifaceted and the Levesque patient‐centred access to healthcare conceptualises the domains to access. Funding policies such as the CDBS may not overcome other barriers such as availability of culturally appropriate services. Tailored, culturally appropriate and evidence‐based models of care utilising the CDBS are urgently needed and should be implemented.

Few children in this study reported access to care in early childhood despite first dental visits being recommended by the age of 2 years. The CDBS legislation has recently been amended to allow eligible children under the age of 2 years to access the schedule (Parliament of Australia, 2021). Access to dental care and preventative oral health education to facilitate early prevention of dental diseases should occur prior to and during the eruption of primary teeth during infancy. Numerous models of care have found early oral health screening by dental and non‐dental professionals to be effective in the prevention of early childhood caries (Heilbrunn‐Lang et al., 2019; Plonka et al., 2013). As the LSAC cohorts were mid‐childhood during the CDBS implementation, future studies should investigate younger cohorts of Australian children who have been eligible to the CDBS during infancy and early childhood and the impact of this schedule on early access to dental services. Further exploration of multi‐disciplinary models of care including screening by nurses, speech pathologists and other early childhood practitioners to encourage early access to dental services is also warranted.

The population weights were another strength of the study, adjusting for participant attrition and allowing population‐level conclusions to be made. The large sample size of the LSAC allowed multiple interactions in a complex statistical model to investigate the research question. Using parental‐reported dental attendance may be limited by recall bias; however, this measure may be the strongest available measure on a population‐level overall dental service use due to the difficulty in measuring attendance in a system dominated by private practice services. Differences between Australian state and territory public dental services may have influenced utilisation, especially in areas with greater utilisation and access to public services such as regional and remote areas. Future studies should investigate utilisation of the schedule amongst private and public sectors to understand utilisation and improve access to the schedule.

## 5 CONCLUSION

This study explored the impact of two Medicare dental schedules reported dental attendance in two cohorts of Australian children. The increase in reported access to dental services and favourable visiting patterns in low‐income households during the operation of the CDBS provides some evidence that the schedule's primary aims to improve access to care in the child population are being met. The lower access to dental care in Aboriginal and Torres Strait Islander children and younger children warrants innovation and policy reform for these populations. Whilst the structure and administration of the CDBS has improved access for sections of the community, there are underserved populations that require urgent additional support to access dental services.

## ETHICS STATEMENT

Ethical approval for the LSAC was granted by the Australian Institute of Family Studies Ethics Committee.

## CONFLICT OF INTEREST

The authors have indicated that they have no potential conflicts of interest to disclose.

## Supporting information

Fig S1

Table S1

## Data Availability

The data that support the findings of this study are available from The Australian Institute of Family Studies (AIFS). Restrictions apply to the availability of these data, which were used under licence for this study. Data are available from the data custodians with the permission of AIFS.
