# Supplementary figures and images for: Has the Child Dental Benefits Schedule improved access to dental care for Australian children?
Source: Health Soc Care Community. 2022 Mar 25;30(6):e4095–102. doi: 10.1111/hsc.13803 (PMC10078627; doi:10.1111/hsc.13803)

Supplementary figure 1.


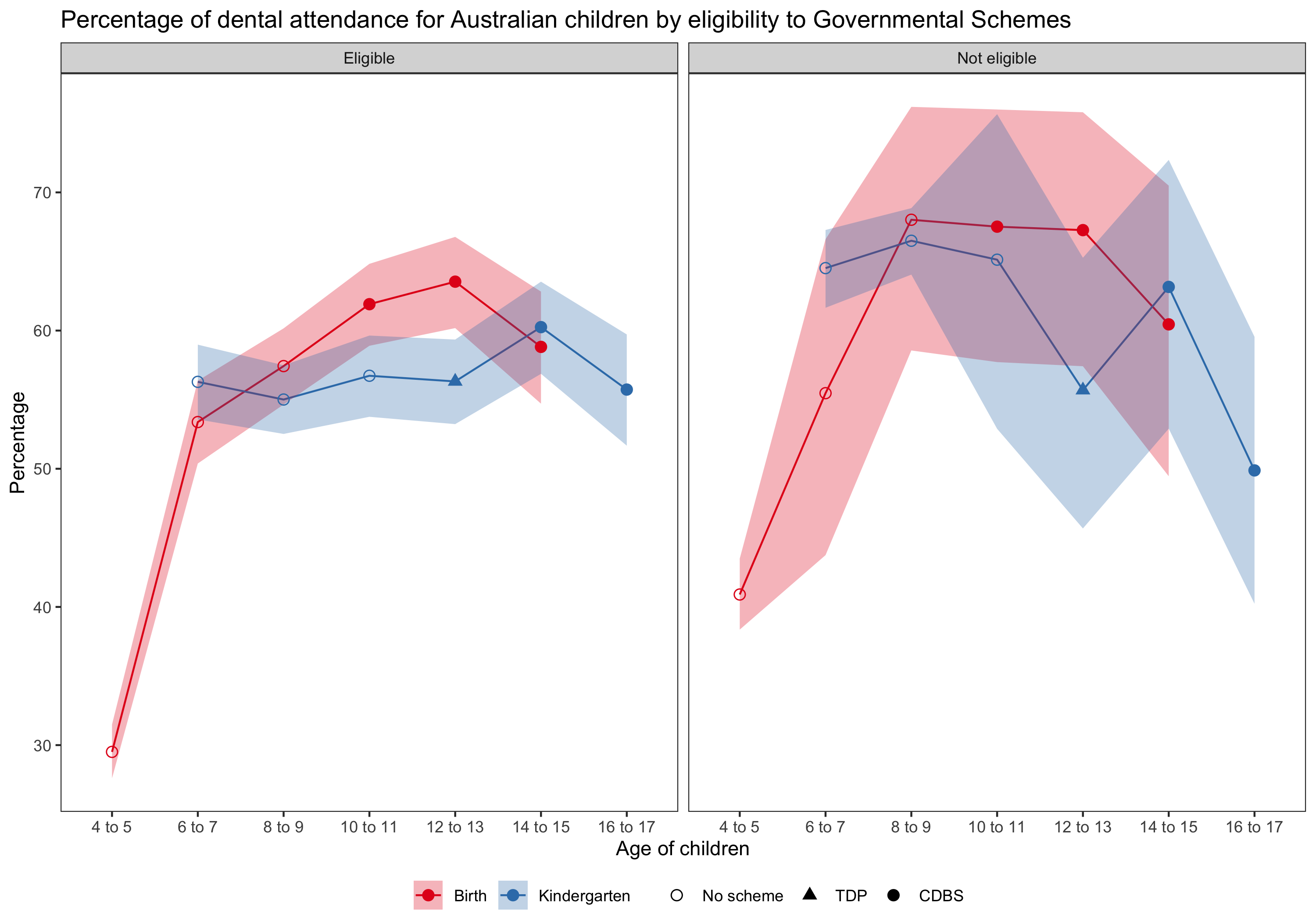

Supplement: Supplementary file 1 — Fig S1 [file HSC-30-e4095-s001.docx]
