# Supplementary material for: Has the Child Dental Benefits Schedule improved access to dental care for Australian children?
Source: Health Soc Care Community. 2022 Mar 25;30(6):e4095–102. doi: 10.1111/hsc.13803 (PMC10078627; doi:10.1111/hsc.13803)
Supplement: Supplementary file 2 — Table S1 [file HSC-30-e4095-s002.docx]

Supplementary table 1. Response rate per wave of the Longitudinal Study of Australian Children (n= 10,090).

| Age (years) | Birth cohort | | | Kindergarten cohort | | |
| --- | --- | --- | --- | --- | --- | --- |
|  | Year | n | Response rate from baseline % | Year | n | Response rate from baseline % |
| 0 to 1 | 2004 | 5107 |  | *na* |  |  |
| 2 to 3 | 2006 | 4606 | 90.2 | *na* |  |  |
| 4 to 5 | 2008 | 4386 | 85.9 | 2004 | 4983 |  |
| 6 to 7 | 2010 | 4241 | 83.0 | 2006 | 4464 | 89.6 |
| 8 to 9 | 2012 | 4085 | 80.0 | 2008 | 4332 | 86.9 |
| 10 to 11 | 2014 | 3764 | 73.7 | 2010 | 4164 | 83.6 |
| 12 to 13 | 2016 | 3381 | 66.2 | 2012 | 3956 | 79.4 |
| 14 to 15 | 2018 | 3127 | 61.2 | 2014 | 3537 | 71.0 |
| 16 to 17 | *na* |  |  | 2016 | 3089 | 62.0 |
| 18 to 19 | *na* |  |  | 2018 | 3037 | 60.9 |

*na*: Not available.

Shading: Light grey Teen Dental Plan Scheme available for eligible children. Dark grey Child Dental Benefits Scheme available for eligible children.
